# Comparative study of *Arabidopsis* PBS1 and a wheat PBS1 homolog helps understand the mechanism of PBS1 functioning in innate immunity

**DOI:** 10.1038/s41598-017-05904-x

**Published:** 2017-07-14

**Authors:** Jianhang Sun, Guozhong Huang, Fenggui Fan, Shuangfeng Wang, Yingying Zhang, Yufang Han, Yanmin Zou, Dongping Lu

**Affiliations:** 1 State Key Laboratory of Plant Genomics, Center for Agricultural Resources Research, Institute of Genetics and Developmental Biology, Chinese Academy of Sciences, Shijiazhuang, Hebei 050021 China; 2 University of Chinese Academy of Sciences, Beijing, 100049 China

## Abstract

*Arabidopsis* AVRPPHB SUSCEPTIBLE1 (PBS1) serves as a “decoy” in activating RESISTANCE TO PSEUDOMONAS SYRINGAE5 (RPS5) upon cleavage by Pseudomonas phaseolicola B (AvrPphB), a *Pseudomonas syringae* effector. The SEMPH motif in PBS1 was thought to allow it to be distinguished by RPS5 from the closely related *Arabidopsis* kinases. However, the underlying mechanism is not fully understood. Here, we isolated and characterized a wheat PBS1 homolog, TaPBS1. Although this plasma membrane-localized kinase could be cleaved by AvrPphB and could associate with RPS5, it failed to trigger RPS5-mediated hypersensitive response (HR) in a transient assay. TaPBS1 harbors a STRPH motif. The association of RPS5 with TaPBS1 was weaker than with PBS1. Change of the STRPH motif to the SEMPH motif allowed TaPBS1 to trigger HR. However, the SEMPH motif is not required for association of PBS1 with RPS5. The difference between “SEMPH” and “STRPH” points to the importance of “EM” in PBS1. Furthermore we found that a negatively charged amino acid at the position of “E” in the SEMPH motif was required for recognition of PBS1 by RPS5. Additionally, both PBS1 and TaPBS1 undergo the flagellin-induced phosphorylation. Therefore, our work will help understand the mechanism of PBS1 functioning in plant innate immunity.

## Introduction

Plants are exposed to an environment full of microorganisms. To defend against attacks from potentially pathogenic microorganisms, plants have evolved sophisticated defense systems, including the preformed non-host defense and plant innate immunity. Plant innate immunity is initiated following pathogen recognition mediated by transmembrane cell-surface receptors and intracellular receptors^1, 2^. The former receptors, referred as pattern recognition receptors (PRRs), perceive conserved pathogen-associated molecular patterns (PAMPs) derived from diverse microorganisms and thus activate immune responses known as PAMP-triggered immunity (PTI)^3^. To successfully colonize the hosts, numerous microorganisms secrete a wide array of effectors to target functional PTI signaling components and suppress PTI^4, 5^. During evolution, plants acquired another perception mechanism: using intracellular disease resistance (R) proteins to detect pathogenic effectors either directly or indirectly and thus activate effector-triggered immunity (ETI). R proteins primarily have a typical NB-LRR structure, with a central nucleotide-binding domain and C-terminal leucine-rich repeats^6, 7^. Generally, ETI is characterized by the hypersensitive response (HR), with localized programmed cell death (PCD) around the pathogen infection site^8^.

Receptor-like protein kinases (RLKs) play important roles in plant innate immunity signaling^9^. The *Arabidopsis* genome encodes more than 610 RLKs. A typical RLK contains an N-terminal extracellular domain, a transmembrane domain (TMD) and a C-terminal protein kinase domain^10^. A number of *Arabidopsis* PRRs, such as FLAGELLIN SENSING 2 (FLS2), EF-Tu receptor (EFR), and CHITIN ELICITOR RECEPTOR KINASE 1 (CERK1), all belong to RLKs, and they recognize the PAMPs bacterial flagellin and its derived peptide flg22, elongation factor Tu (EF-Tu) and its derived peptide elf18, and the fungal cell wall component chitin, respectively^11–14^. Following PAMP recognition, both FLS2 and EFR associate with another RLK, BAK1, to transduce immune signaling^15, 16^.

Plant receptor-like cytoplasmic kinases (RLCKs) are a subset of RLK family members. They lack both extracellular and transmembrane domains but possess kinase domains that are homologous to those of the typical RLKs^17^. An increasing number of RLCKs have been shown to play important roles in plant innate immunity. AVRPPHB Susceptible1 (PBS1) and a number of PBS1-like (PBL) proteins, such as BIK1, PBL1, and PBL2, all from the RLCKs VII subfamily, associate with FLS2 and transduce immune signaling from the cell surface immune receptors^18, 19^. Flagellin induces phosphorylation of these RLCKs^19^. BIK1 directly phosphorylates the NADPH oxidase RbohD at specific sites, thus to control ROS generation and stomatal immunity^20, 21^. The mutant plants of *bik1*, *pbl1*, *pbl2*, and *pbs1* are all compromised to varying degrees in defense responses. However, compared with *bik1*, *pbs1* mutant exhibits only marginal defects in PTI defenses^19^.

As key components of PTI signaling, certain immune-related RLCKs serve as targets for pathogen effectors. For example, BIK1 is targeted by AvrAC, an effector from *Xanthomonas campestris* pathovar *campestris* (*Xcc*)^22^. Additionally, BIK1, PBL1, PBL2, PBS1 and other PBL proteins could be cleaved by the *Pseudomonas syringae* effector Avirulence protein Pseudomonas phaseolicola B (AvrPphB), which functions as a cysteine protease in host cells^19, 23^. AvrPphB likely targets and cleaves these kinases to inhibit plant immune responses^19^. Although a number of RLCKs could be cleaved by AvrPphB, only the cleavage of PBS1 by AvrPphB is detected by the R protein RESISTANCE TO PSEUDOMONAS SYRINGAE5 (RPS5) to activate ETI responses^23, 24^. Therefore, PBS1 may serve as a “decoy” during ETI by mimicking true virulence targets, such as BIK1^19^. PBS1 is localized to the plasma membrane via N-terminal *S*-acylation^24^, where it associates with the N-terminal coiled-coil (CC) domain of RPS5^25^. It was proposed that the SEMPH motif in a C-terminal loop of PBS1 specifically allows PBS1 to be distinguished by RPS5 from the closely related *Arabidopsis* RLCKs^24^. However, the mechanism underlying the requirement of the PBS1 SEMPH motif in RPS5 activation is not fully understood. A comparative study of a gene and its homolog from other species may help better understand its functioning mechanism. Here, we isolated and characterized TaPBS1, a PBS1 homolog from wheat (*Triticum aestivum* cv. Kn9204), and performed a comparative study between PBS1 and TaPBS1. Despite the ability of TaPBS1 to be cleaved by AvrPphB and to associate with the RPS5 CC domain, TaPBS1 failed to trigger RPS5-mediated HR when expressed together with AvrPphB and RPS5 in a transient assay. Unlike PBS1, TaPBS1 has a STRPH motif instead of the SEMPH motif in the corresponding region. Introduction of the SEMPH motif into TaPBS1 resulted in activation of RPS5-mediated HR. However, the SEMPH motif in PBS1 is not required for its association with RPS5. Because the only difference between the SEMPH motif and the STRPH motif is the “EM” versus “TR”, our work points to the importance of “EM” in the SEMPH motif. Furthermore, by HR assays in *pbs1* mutant protoplasts, we found that a negatively charged amino acid at the position of “E” in the SEMPH motif is required for recognition of PBS1 by RPS5. Therefore, this work helps understand the mechanism of PBS1 functioning as a “decoy”.

## Results

### TaPBS1 has a STRPH motif instead of the SEMPH motif

**Figure 1:**
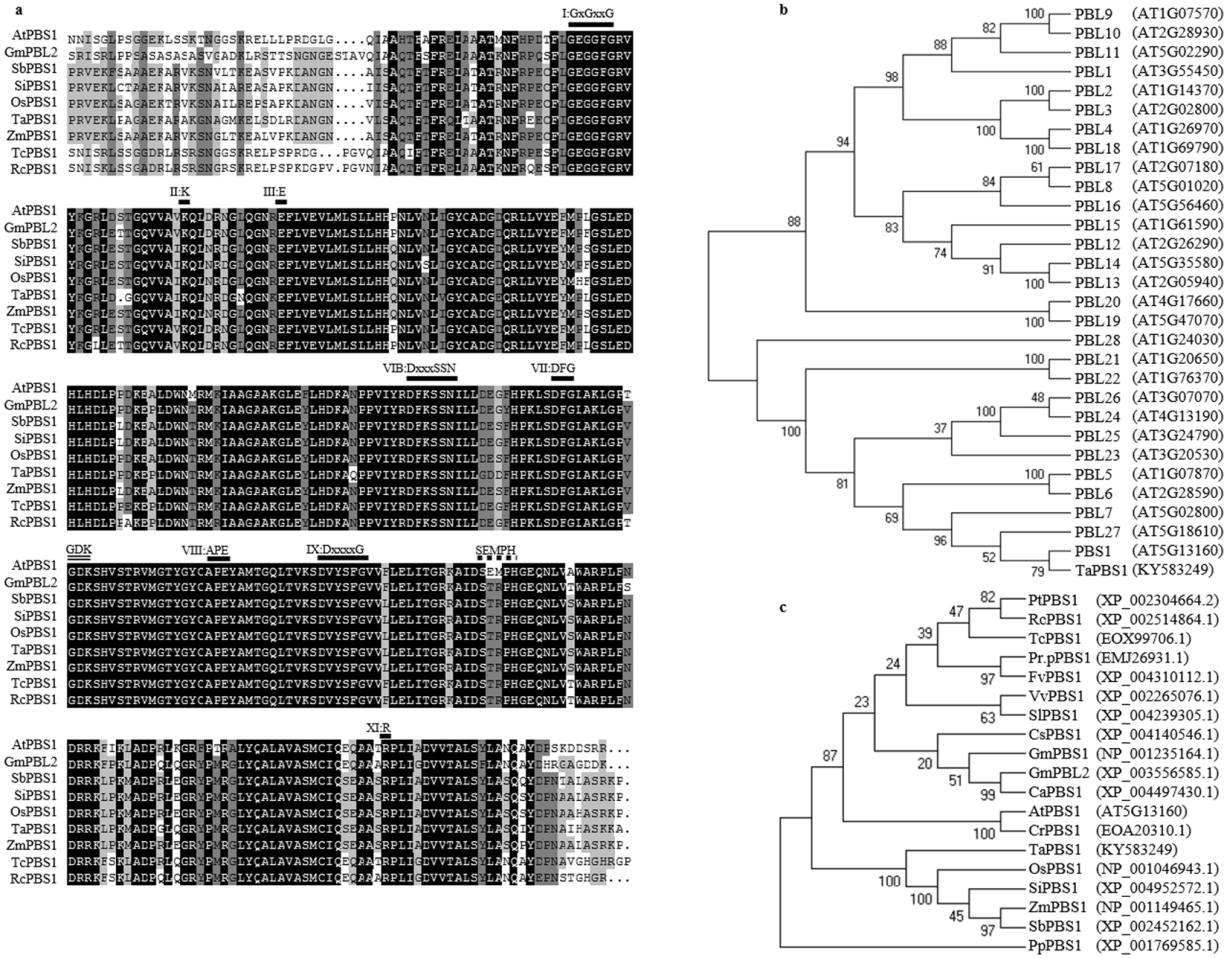
Phylogenetic analysis and multiple alignment of TaPBS1 and related proteins. (**a**) Amino acid sequence alignment of TaPBS1 with putative PBS1 orthologs from different plant species. Identical and similar amino acid residues are shown on black and gray backgrounds, respectively. The conserved kinase subdomains are labeled with Roman numbers above the aligned sequences. The AvrPphB cleavage site GDK and the SEMPH/STRPH motif are also labeled. (**b**) Phylogenetic analysis of TaPBS1 and *Arabidopsis* PBS1 paralogs. A neighbor-joining phylogenetic tree was constructed based on the deduced amino acid sequences of TaPBS1, PBS1 and 29 PBL proteins in *Arabidopsis* using MEGA5.0.3 software. (**c**) Phylogenetic analysis of PBS1 orthologs from different plant species. *Ca*: *Cicer arietinum*; *Gm*: *Glycine max*; *Cs*: *Cucumis sativus*; *Pt*: *Populus trichocarpa*; *Rc*: *Ricinus communis*; *Tc*: *Theobroma cacao*; *Pr*.*p*: *Prunus persica*; *Fv*: *Fragaria vesca*; *Vv*: *Vitis vinifera*; *Sl*: *Solanum lycopersicum*; *At*: *Arabidopsis thaliana*; *Cr*: *Capsella rubella*; *Pp*: *Physcomitrella patens*; *Ta*: *Triticum aestivum*; *Os*: *Oryza sativa*; *Si*: *Setaria italica*; *Zm*: *Zea mays*; *Sb*: *Sorghum bicolor*.

To perform a comparative study between *PBS1* and its homolog gene from higher plant species, we isolated *TaPBS1* (NCBI accession NO. KY583249) from wheat (*Triticum aestivum* cv. Kn9204). TaPBS1 contains a single catalytic kinase domain as defined by the presence of conserved amino acid residues^26^ (Fig. 1a). TaPBS1 showed the closest relationship with AtPBS1 (sharing 66% amino acid sequence identity and 75% similarity) among PBS1 and 29 *Arabidopsis* PBL proteins in a phylogenetic tree analysis using the Neighbor-Joining method implemented in MEGA5.0.3 (Fig. 1b). The phylogenetic analysis of PBS1 homologs from various species was performed using the moss PBS1 homolog PpPBS1 as an outgroup. The results showed that PBS1 homologs from monocotyledons, such as wheat, rice, millet, maize and sorghum, were clustered into a clade. *Arabidopsis* PBS1 and other dicotyledon PBS1 homologs formed another clade (Fig. 1c). OsPBS1 is the protein with the highest similarity to TaPBS1; they share 78% amino acid similarity and 73% amino acid identity. In addition to *TaPBS1*, wheat genome should have other *PBS1* homologs since wheat is an allohexaploid. For example, two progenitor species of hexaploid wheat, *Triticum Urartu* and *Aegilops tauschii*, each diploid species has a *PBS1* homolog, *TuPBS1* or *AetPBS1* (Supplementary Fig. S1).

### Cleavage of TaPBS1 by AvrPphB could not activate *Arabidopsis* RPS5

PBS1 and TaPBS1 have independently evolved after the separation of monocots and dicots. Usually, different plant species have distinct but sometimes overlapping pathogens. The effectors secreted by pathogens evolve rapidly, driving plants to have evolved a resistance system with great complexity^27, 28^. Therefore, TaPBS1 is theoretically unlikely to have the function to activate *Arabidopsis* RPS5. As expected, no HR was observed when TaPBS1 was expressed together with AvrPphB and RPS5 in *Nicotiana benthamiana* (Supplementary Fig. S2a,b). Then we were curious to identify the difference between PBS1 and TaPBS1 that accounts for the failure of TaPBS1 to activate RPS5.

**Figure 2:**
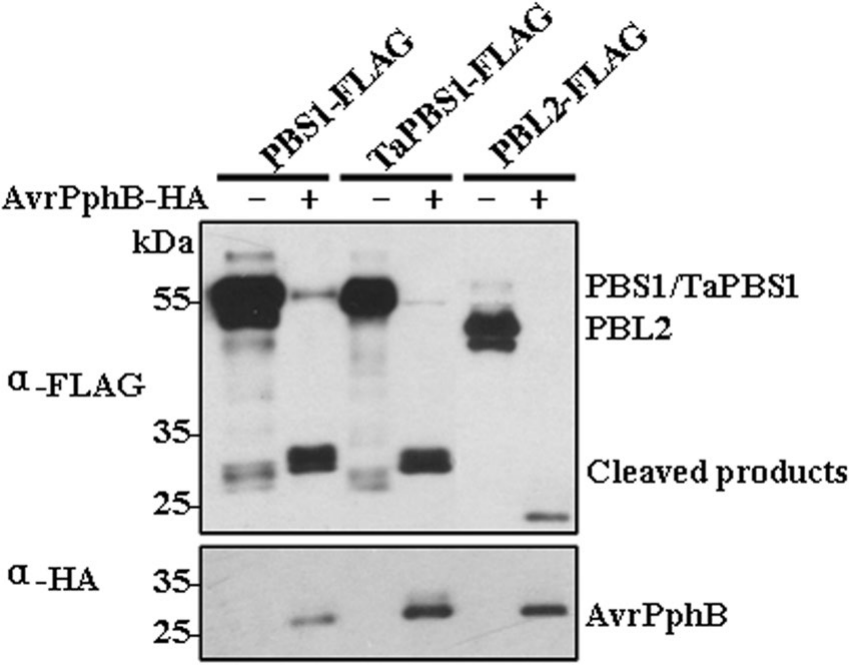
Cleavage of TaPBS1 by AvrPphB in *Arabidopsis* protoplasts. TaPBS1 could be cleaved by AvrPphB. FLAG-tagged PBS1, TaPBS1, or PBL2 was expressed together with AvrPphB-HA in *Arabidopsis* protoplasts. Protein cleavage was detected by Western blot analysis with an anti-FLAG antibody. AvrPphB-HA was detected by Western blot analysis with an anti-HA antibody.

Firstly, we would like to ask whether TaPBS1 could be cleaved by AvrPphB. *Arabidopsis* PBS1 is cleaved by AvrPphB right after the GDK motif, thus activating RPS5^23^. Interestingly, TaPBS1 also has a GDK motif (Fig. 1a). We found that, similar to PBS1 and PBL2, TaPBS1 could also be cleaved by AvrPphB when they were co-expressed in *Arabidopsis* protoplasts or *N*. *benthamiana*, suggesting that failure of TaPBS1 to activate RPS5 is not because TaPBS1 could not be cleaved by AvrPphB (Figs 2 and S2b).

### TaPBS1 associates with the CC domain of RPS5

**Figure 3:**
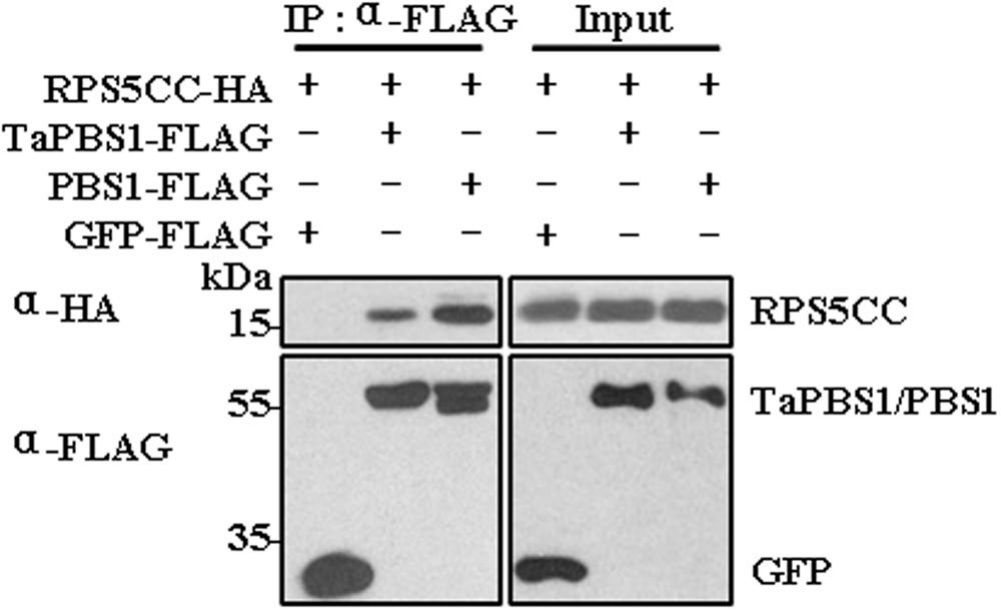
TaPBS1 associates with the CC domain of RPS5. TaPBS1/PBS1-FLAG and RPS5-CC-HA were co-expressed in *Arabidopsis* protoplasts, and TaPBS1/PBS1 was precipitated with an anti-FLAG antibody. The associated proteins were analyzed by Western blotting with an anti-HA antibody. GFP-FLAG was used as a negative control.

*Arabidopsis* PBS1 forms a complex with the CC domain of RPS5 (RPS5CC) prior to AvrPphB exposure^25^. To rule out the possibility that failure of TaPBS1 to activate RPS5 is due to its inability to associate with the CC domain of RPS5, we tested whether TaPBS1 is physically associated with RPS5CC using a co-IP assay. TaPBS1 and the RPS5 CC domain were co-expressed in *Arabidopsis* protoplasts, and the results showed that TaPBS1 could associate with the CC domain of RPS5, however the association was weaker than that of PBS1 with RPS5CC under this assay condition (Fig. 3). In addition, the RPS5 CC domain failed to associate with GFP-FLAG, suggesting that RPS5CC binds to PBS1/TaPBS1, instead of FLAG-tag (Fig. 3).

### TaPBS1 is localized to the plasma membrane

**Figure 4:**
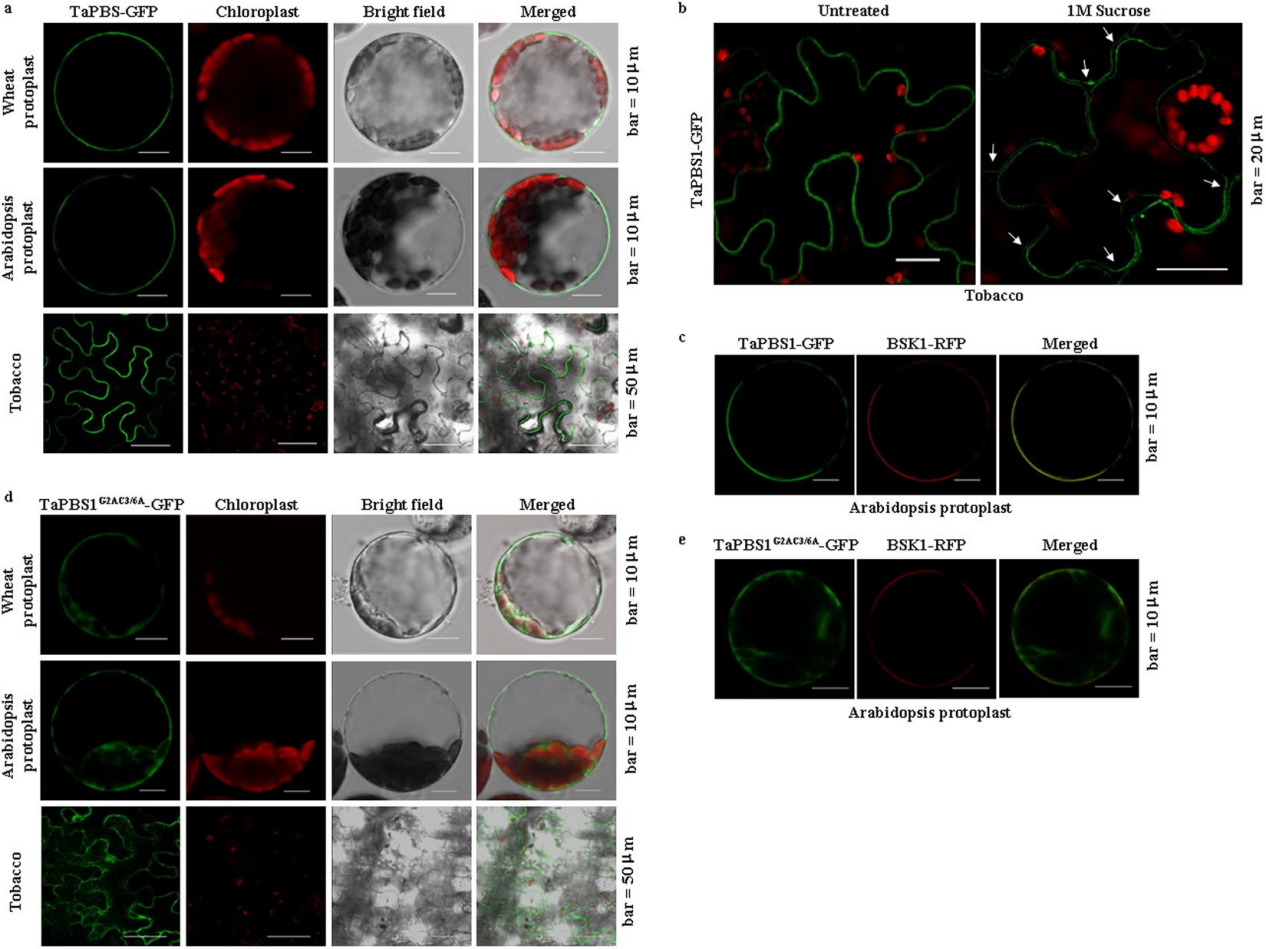
TaPBS1 is localized to the plasma membrane. (**a**) The subcellular localization of TaPBS1 in *Arabidopsis* protoplasts, wheat protoplasts, and *N*. *benthamiana* leaves. TaPBS1 was transiently expressed in wheat protoplasts, *Arabidopsis* protoplasts or *N*. *benthamiana* leaves, and then subjected to laser confocal imaging. Chloroplasts were visualized with the autofluorescence of chlorophyll. (**b**) The plasmolyzed *N*. *benthamiana* epidermal cells expressing TaPBS1-GFP (arrows) demonstrated the PM localization of TaPBS1. *N*. *benthamiana* leaves infiltrated with *A*. *tumefaciens* carrying the *TaPBS1-GFP* construct were mounted in 1 M sucrose. (**c**) TaPBS1-GFP is co-localized with BSK1-RFP, a PM-localized fusion protein, at the PM. (**d**) Alanine substitutions of predicted myristoylation (glycine-2) and palmitoylation (cysteine-3/6) residues affected the PM localization of TaPBS1. TaPBS1^G2AC3/6A^-GFP was transiently expressed in wheat protoplasts, *Arabidopsis* protoplasts or *N*. *benthamiana* leaves. (**e**) Co-localization analysis of TaPBS1^G2AC3/6A^-GFP and BSK1-RFP.

PBS1 has an N-terminal *S*-acylation signal (MG**C**FS**C**FDS), with both Cys-3 and Cys-6 residues palmitoylated, which enables PBS1 to localize to the plasma membrane (PM). The PM localization of PBS1 is required for RPS5-mediated resistance in *Arabidopsis* ^24^. The corresponding region in TaPBS1 (MGCFPCFDS) is very similar to that of PBS1, with only a difference in the 5th amino acid. To rule out the possibility that inability of TaPBS1 to activate RPS5 is due to its subcellular localization, we tested whether TaPBS1 is also localized to the PM or not. We transiently expressed TaPBS1-GFP in wheat protoplasts, *Arabidopsis* protoplasts, and *N*. *benthamiana* epidermal cells. Confocal laser scanning microscopy imaging revealed that TaPBS1 was localized to the PM in all three plant species (Fig. 4a). Especially, the plasmolyzed *N*. *benthamiana* epidermal cells expressing TaPBS1-GFP confirmed the PM localization of TaPBS1 (Fig. 4b). To further verify the PM localization of TaPBS1, we co-expressed TaPBS1-GFP together with BSK1-RFP, a PM-localized RLCK fused to a red fluorescence protein tag in *Arabidopsis* protoplasts^29^. The results showed that TaPBS1-GFP was co-localized with BSK1-RFP at the PM, indicating that TaPBS1 is indeed localized to the PM (Fig. 4c).

To confirm that the PM localization of TaPBS1 was also mediated by *S*-acylation, we analyzed the localization of a TaPBS1 variant with the G2AC3/6A mutation. We found that Alanine substitutions of predicted myristoylation (glycine-2) and palmitoylation (cysteine-3/6) residues affected the PM localization of TaPBS1. However, it seems that some portion of TaPBS1^G2AC3/6A^ proteins are still localized to the PM (Fig. 4d). Furthermore, we co-expressed TaPBS1^G2AC3/6A^-GFP together with BSK1-RFP in *Arabidopsis* protoplasts, the results demonstrated that the majority of TaPBS1^G2AC3/6A^-GFP was no longer co-localized with BSK1-RFP at the PM (Fig. 4e). These results suggest that the PM localization of TaPBS1 is dependent on an N-terminal *S*-acylation signal.

### TaPBS1 has kinase activity *in vitro*

**Figure 5:**
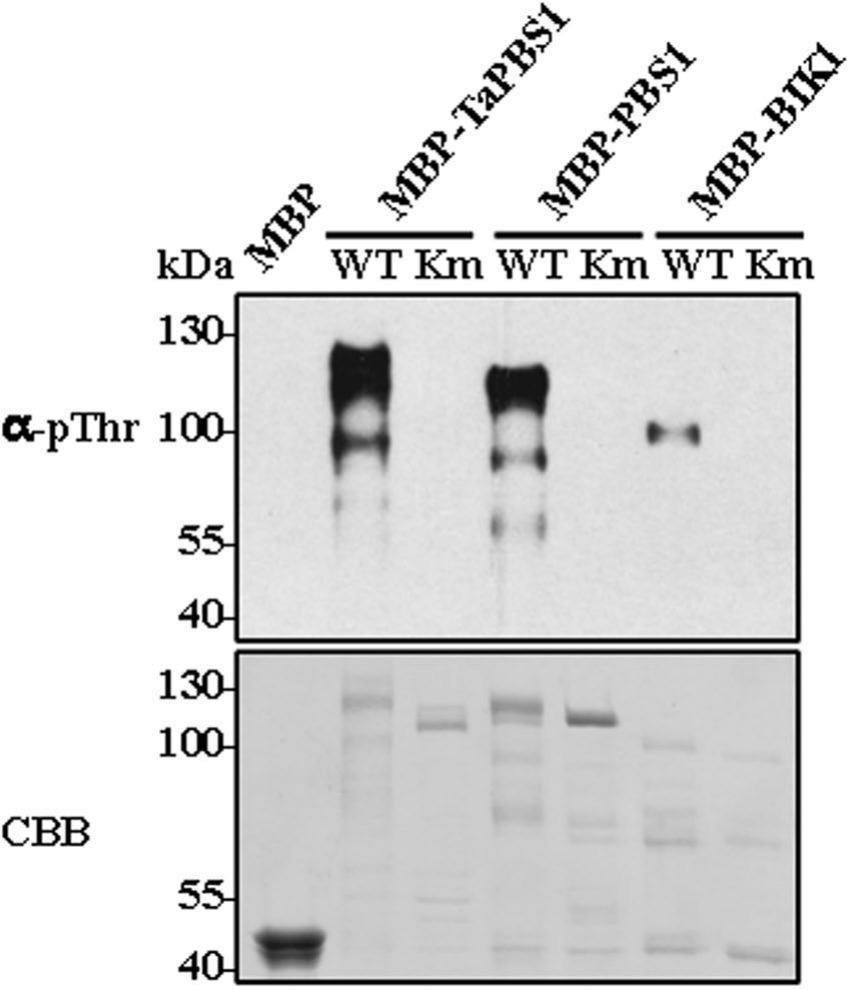
*In vitro* analysis of TaPBS1 autophosphorylation. An *in vitro* protein phosphorylation assay was performed by incubating MBP, MBP-TaPBS1, MBP-TaPBS1Km (K132A), MBP-PBS1, MBP-PBS1Km (K115N), MBP-BIK1, and MBP-BIK1Km (K105A) in the presence of ATP. Proteins were separated with SDS-PAGE, and the protein phosphorylation was detected by Western blot analysis with an anti-phospho-Thr (pThr) antibody (upper panel). The protein loading control was shown by Coomassie brilliant blue staining (CBB, lower panel).

The PBS1 kinase activity is required for its functioning in activating RPS5 in *Arabidopsis* under native protein levels^23^, then we tested whether TaPBS1 has kinase activity *in vitro*. TaPBS1 contains a single catalytic kinase domain as defined by the presence of conserved amino acid residues^26^ (Fig. 1a). Also, a conserved lysine (K132) residue lies in a region within the kinase subdomian II predicted to be essential for ATP binding (Fig. 1a). Kinases can generally undergo autophosphorylation in the presence of ATP, and this feature was used to assay the kinase activity. Both PBS1 and BIK1 have autophosphorylation activity, while PBS1Km(K115N) and BIK1Km(K105A) that are mutated in the ATP-binding sites, lack kinase activity^18, 23^. To analyze the kinase activity of TaPBS1, we performed an *in vitro* kinase assay with the purified recombinant MBP-TaPBS1 proteins. Similar to PBS1 and BIK1, TaPBS1 showed strong autophosphorylation activity as detected by an anti-phosphotheronine antibody, while TaPBS1Km(K132A), which had a deficiency in ATP binding, did not (Fig. 5). These results indicate that at least certain threonine residues on both TaPBS1 and PBS1 are autophosphorylated *in vitro*.

### Change of the STRPH motif to the SEMPH motif allows TaPBS1 to trigger RPS5-mediated HR

**Figure 6:**
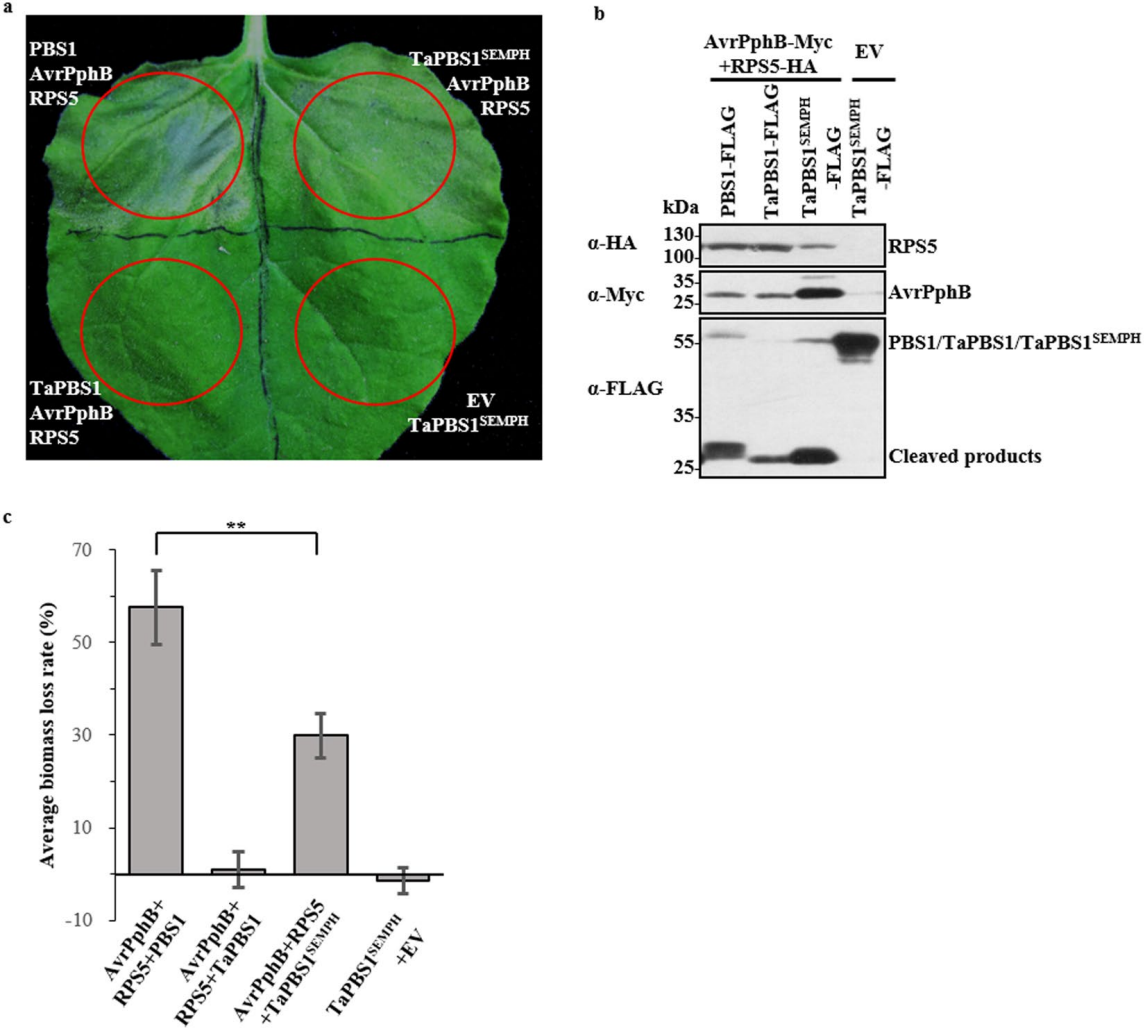
The introduction of the SEMPH motif into TaPBS1 enables it to activate RPS5 in transient assay. (**a**) The replacement of the STRPH motif with the SEMPH motif in TaPBS1 resulted into the RPS5-mediated HR in *N*. *benthamiana* following the cleavage of TaPBS1^SEMPH^ by AvrPphB. TaPBS1 or TaPBS1^SEMPH^ was co-expressed with RPS5 and AvrPphB in *N*. *benthamiana* leaves. HR assays were performed as above. (**b**) The protein expression control for the above assays. (**c**) Quantification of HRs induced in *N*. *benthamiana* leaves. HR was quantified by measuring the biomass loss rate of *N*. *benthamiana* leaves infiltrated with *A*. *tumefaciens* carrying indicated constructs. Results are the means ± SD of three independent repeats. Statistical significance was determined using a Student’s *t*-test: **P < 0.01.

The PM-localized TaPBS1 was associated with the RPS5 CC domain and could be cleaved by AvrpPphB as well. However, it could not activate RPS5-mediated HR when transiently expressed in *N*. *benthamiana*. It was proposed that the SEMPH motif in PBS1 specifically allows *Arabidopsis* PBS1 to be distinguished by RPS5^24^. TaPBS1 has a STRPH motif in the corresponding region which is shared with a vast array of PBS1 homologs from different plant speicies, such as OsPBS1 (rice), ZmPBS1 (maize), SbPBS1 (sorghum), SiPBS1 (millet) and TcPBS1 (cocoa) (Fig. 1a). In addition to the standard STRPH motif, other PBS1 homologs, such as FvPBS1 (strawberry), SlPBS1 (tomato) and VvPBS1 (grape), have a STRPH-like motif: S(G)T(S/N)R(/K/Q/L)P(S)H(Q)^24^. Thus, the SEMPH motif is only specific to *Arabidopsis* PBS1 and the closely related mustard species^24^. Then, we changed the STRPH motif to the SEMPH motif in TaPBS1 and performed an HR assay in *N*. *benthamiana*, where TaPBS1^SEMPH^ was co-expressed together with AvrPphB and RPS5. The results showed that introduction of SEMPH enabled TaPBS1 to activate RPS5 when TaPBS1^SEMPH^ was cleaved by AvrPphB (Fig. 6a,b). However, the RPS5-mediated HR activated by TaPBS1^SEMPH^ is weaker than that by *Arabidopsis* PBS1 as quantified by the biomass loss of the plant tissues (Fig. 6c).

### The SEMPH motif is not required for the association of PBS1 with RPS5

As introduction of SEMPH motif into TaPBS1 could enable RPS5-mediated HR, also the association of TaPBS1 and RPS5CC was weaker than that of PBS1 with RPS5CC (Fig. 3), then we asked whether the SEMPH motif is required for the association of PBS1 with the CC domain of RPS5. The results of Co-IP assays showed that, although the association of TaPBS1 with RPS5CC was relatively weaker than that of PBS1 with RPS5CC, introduction of the SEMPH motif into TaPBS1 could not enhance its association with the RPS5 CC domain, similarly, the change of the SEMPH motif to the STRPH motif in PBS1 did not reduce its association with RPS5CC (Supplementary Fig. S3). These results suggest that, although recognition of PBS1 cleavage relies on the SEMPH motif, the SEMPH motif is not required for the association of PBS1 to the CC domain of RPS5. Hence, the relatively weaker association of TaPBS1 with RPS5 could be caused by other factors rather than the STRPH motif of TaPBS1, and the binding of PBS1 to RPS5 was through other unknown amino acid residues in PBS1.

### A negatively charged amino acid at the position of “E” in the SEMPH motif is required for the recognition of PBS1 by RPS5

**Figure 7:**
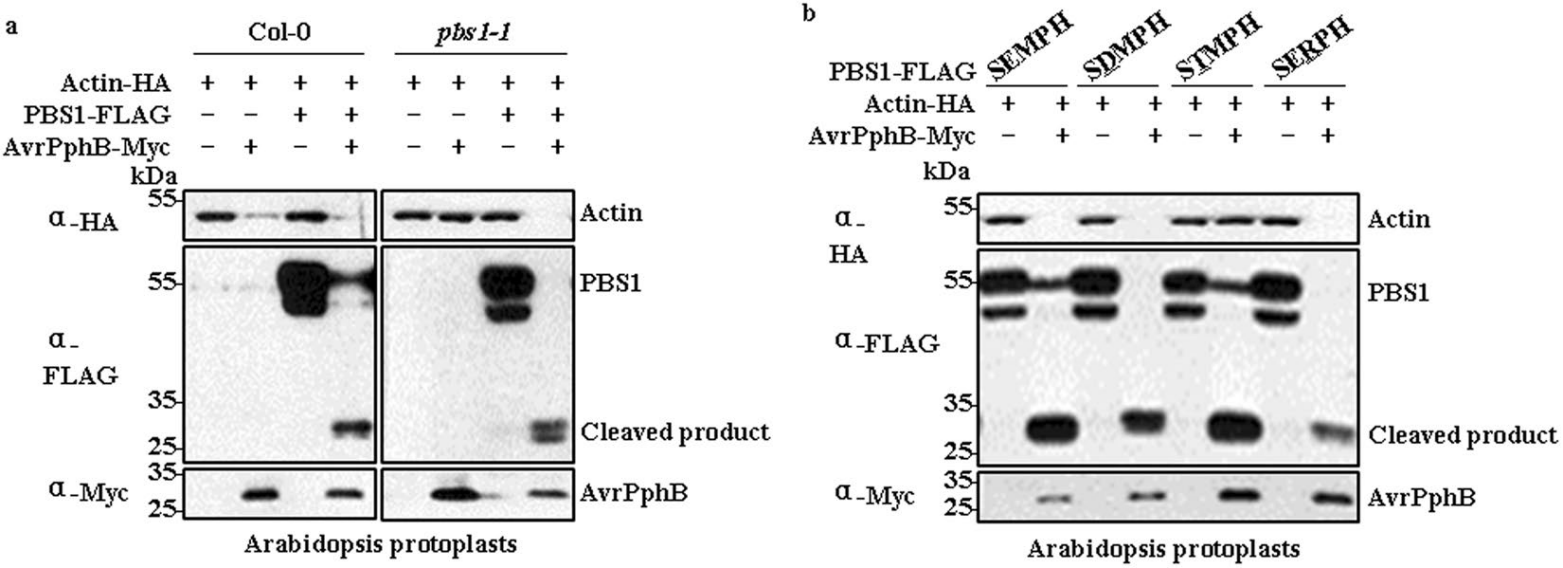
A negatively charged amino acid at the position of “E” in the SEMPH motif is required for recognition of PBS1 by RPS5. (**a**) Development of an AvrPphB-induced PCD assay system in *pbs1* protoplasts. AvrPphB was co-expressed together with PBS1 and Actin-HA in *Arabidopsis* mesophyll protoplasts isolated from *pbs1* plants. The PCD induced in *Arabidopsis* mesophyll protoplasts was monitored by the repression of Actin-HA expression. (**b**) A negatively charged amino acid at the position of “E” in the SEMPH motif is required for recognition of PBS1 by RPS5, while the amino acid “M” may not be essential for the recognition. Three PBS1 mutants were generated with either “E” or “M” mutated. Then each PBS1 mutant was expressed together with AvrPphB and Actin-HA in *pbs1* protoplasts.

Because the only difference between the SEMPH motif and the STRPH motif is “EM” versus “TR”, our work points to the importance of “EM” in the SEMPH motif. To test whether both “E” and “M” are required for recognition by RPS5, we deployed a plant mesophyll protoplast system in which AvrPphB is transiently expressed to induce HR-related PCD. It was previously reported that expression of effector genes, like *AvrRpm1*, *AvrB* or *AvrRpt2*, in *Arabidopsis* protoplasts triggered distinct kinetics of PCD as detected by Evan’s blue staining^30^. The cell death in protoplasts could also be evaluated by quantification of GUS activity repression when UBQ10-GUS was expressed together with an effector in *Arabidopsis* protoplasts^30^. Here, we co-transfected *AvrPphB* together with *Actin-HA* in *Arabidopsis* mesophyll protoplasts isolated from either col-0 or *pbs1* plants. We found that transient expression of AvrPphB could induce PCD in col-0 but not in *pbs1*, as demonstrated by the repression of Actin-HA expression. However, co-transfection of *AvrPphB* together with *PBS1* in *pbs1* protoplasts could trigger PCD (Fig. 7a). Therefore we developed a HR assay system in *pbs1* protoplasts that could be used to identify critical sites in PBS1 for its recognition by RPS5. And this system could also be applied to “decoy engineering” to quickly test whether the engineered PBS1 proteins could trigger HR upon specific cleavage by effector proteases, prior to generating stable transgenic plants^31, 32^.

Here, to test whether both “E” and “M” amino acid residues in the SEMPH motif are required for recognition by RPS5, we generated three PBS1 mutants with either “E” or “M” mutated. Then each PBS1 mutant was expressed together with AvrPphB and Actin-HA in *pbs1* protoplasts. When the SEMPH motif was changed to SDMPH, where “E” was replaced by another negatively charged amino acid “D”, expression of AvrPphB together with PBS1^SDMPH^ could still induce cell death in *pbs1* protoplasts. However, when “E” was changed to “T”, the corresponding amino acid residue in the STRPH motif, PBS1^STMPH^ failed to function in AvrPphB-induced cell death. Interestingly, when “M” was substituted by “R”, the corresponding residue in the STRPH motif, PBS1^SERPH^ still worked well in AvrPphB-induced cell death (Fig. 7b). These results suggest that a negatively charged amino acid at the position of “E” in the SEMPH motif is required for the recognition of PBS1 by RPS5, while the amino acid “M” may not be essential for the recognition.

### Flagellin peptide induces TaPBS1 phosphorylation *in vivo*

**Figure 8:**
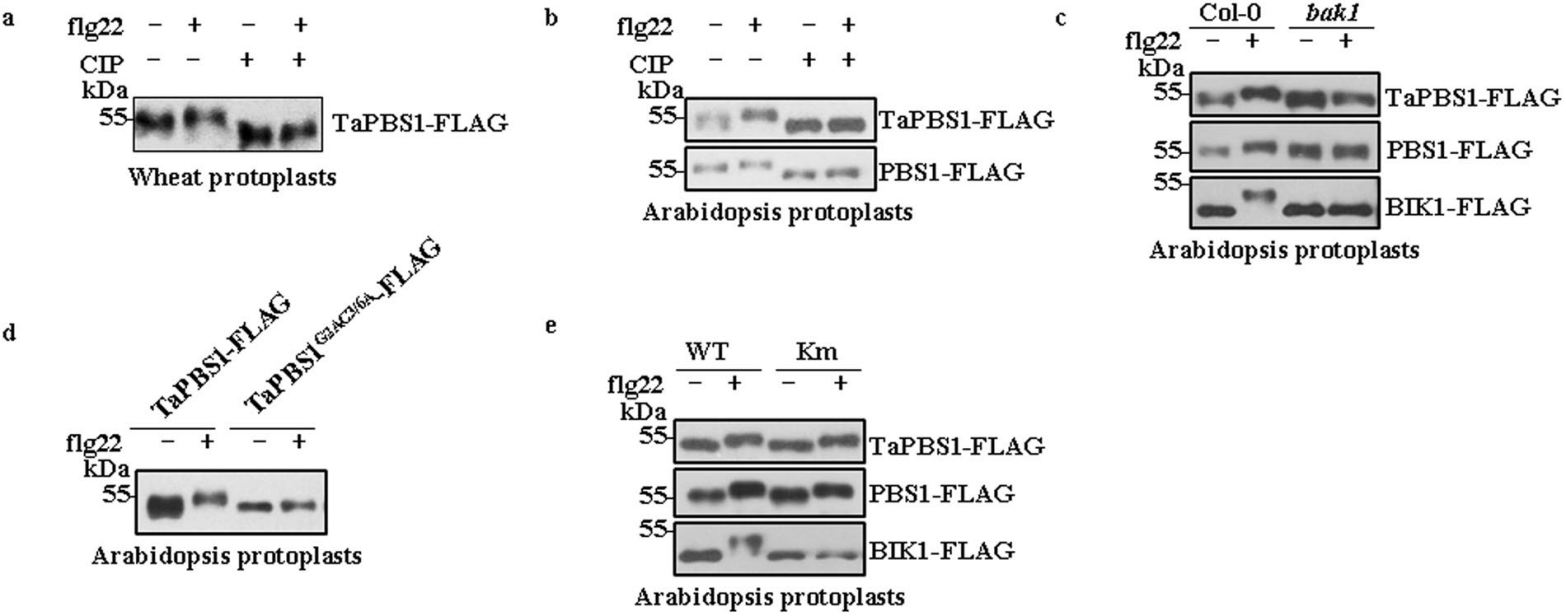
Flagellin induces TaPBS1 phosphorylation *in vivo*. (**a**) Flg22 induces a mobility shift of TaPBS1 expressed in wheat protoplasts. TaPBS1-FLAG was transfected in wheat protoplasts. Then the wheat protoplasts were incubated for 8 h and treated with 1 μM flg22 for 10 min before harvesting. For CIP (calf intestinal alkaline phosphatase) treatment, 1 μM CIP was applied in the reactions containing TaPBS1 proteins and incubated at 37 °C for 1 h. Proteins were separated with SDS-PAGE, and TaPBS1 was detected by Western blot analysis with an anti-FLAG antibody. (**b**) Flg22 induces a mobility shift of TaPBS1 expressed in *Arabidopsis* protoplasts. TaPBS1 or PBS1 was expressed in *Arabidopsis* protoplasts and was analyzed as above. (**c**) The flg22-induced TaPBS1 phosphorylation in *Arabidopsis* protoplasts depends on BAK1. Protoplasts were isolated from col-0 or *bak1* mutant plants and were transfected with FLAG-tagged *TaPBS1*, *PBS1*, or *BIK1*. (**d**) The flg22-induced TaPBS1 phosphorylation in *Arabidopsis* protoplasts depends on its PM localization. *Arabidopsis* protoplasts were transfected with *TaPBS1* or *TaPBS1* ^*G2AC3*/*6A*^ and treated with 1 μM flg22 for 10 min before harvesting. The protein mobility shift was analyzed by Western blotting. (**e**) The kinase activity is not required for the flg22-induced TaPBS1/PBS1 phosphorylation. *Arabidopsis* protoplasts were transfected with *TaPBS1*, *TaPBS1Km*, *PBS1*, *PBS1Km*, *BIK1* or *BIK1Km* and were treated with 1 μM flg22 for 10 min before harvesting.

Although TaPBS1 failed to activities *Arabidopsis* RPS5 upon cleavage by AvrPphB, we want to ask whether they share some PTI-related characteristics. It has been shown that flg22 can induce phosphorylation of both PBS1 and BIK1, as exhibited by protein mobility shifts on SDS-PAGE, suggesting their involvement in PTI signaling^18, 19, 33^. Therefore, we assessed whether flg22 can induce TaPBS1 phosphorylation *in vivo*. When FLAG epitope-tagged TaPBS1 was expressed in wheat protoplasts, the flg22 treatment induced a mobility shift of TaPBS1 protein on SDS-PAGE as detected by Western blotting (Fig. 8a). To confirm that this shift is indeed caused by phosphorylation, we treated the TaPBS1 proteins expressed in wheat protoplasts with calf alkaline intestinal phosphatase (CIP). The results showed that after CIP treatment, the flg22-induced mobility shift of TaPBS1 was not observed. Notably, following CIP treatment, the migration of TaPBS1 was overall faster than that of untreated samples, suggesting that TaPBS1 may also have undergone phosphorylation on sites other than those phosphorylated upon flg22 treatment (Fig. 8a). Interestingly, the flg22-induced TaPBS1 phosphorylation also occurred in *Arabidopsis* protoplasts (Fig. 8b). This suggests that the signaling pathways upstream of PBS/TaPBS1 may be conserved to some extents between *Arabidopsis* and wheat. BAK1 serves as the signaling partner of the flg22 receptor FLS2. To test whether flg22-induced TaPBS1 phosphorylation in *Arabidopsis* protoplasts depends on BAK1, we expressed the TaPBS1 in protoplasts isolated from *bak1* mutant plants and found that the flg22-induced phosphorylation of TaPBS1 was not observed in *bak1*, similar to that of BIK1 and PBS1 (Fig. 8c).

As TaPBS1 is localized to the PM, Alanine substitutions of predicted myristoylation (glycine-2) and palmitoylation (cysteine-3/6) residues affected the PM localization of TaPBS1. Then we examined whether the PM localization is required for its flg22-induced phosphorylation. We expressed TaPBS1^G2AC3/6A^ in *Arabidopsis* protoplasts. The results showed that flg22 almost did not induce phosphorylation of this TaPBS1 mutant form (Fig. 8d). However, we have shown that some portion of TaPBS1^G2AC3/6A^ may still retain a peripheral localization (Fig. 4d,e). This discrepancy could be explained by the assumption that some cytoplasmic TaPBS1^G2AC3/6A^ proteins were squeezed out to the cell edge by vacuole and thus show the PM-like localization. But we cannot exclude the possibility that there are other unknown reasons for this phenomenon.

As TaPBS1 can undergo autophosphorylation and, thus, has kinase activity *in vitro*, we also tested whether its kinase activity is required for the flg22-induced phosphorylation. We analyzed the phosphorylation of TaPBS1Km, BIK1Km, and PBS1Km, which all carry mutations in the ATP-binding sites. We found that when kinase activity of BIK1 was abolished, its phosphorylation upon flg22 treatment disappeared. However, either PBS1 or TaPBS1 still showed the flg22-induced phosphoryaltion when its ATP binding site had mutation (Fig. 8e). This indicated that the flg22-induced PBS1/TaPBS1 phosphorylation is likely mediated by other kinases.

The flg22-induced TaPBS1 phosphorylation prompts us to assess whether expression of TaPBS1 affects the flg22-induced PTI outputs in a transient expression system. We co-expressed *TaPBS1*/*PBS1* or *BIK1* together with *pFRK1-LUC*, a luciferase reporter driven by the promoter of a PAMP-responsive gene *FRK1*, in *Arabidopsis* protoplasts. Consistently, we found that expression of BIK1 moderately, but significantly, activated *pFRK1*-*LUC* ^18^, however, neither PBS1 nor TaPBS1 could affect the promoter activity of *FRK1* (Supplementary Fig. S4). Similar results were obtained when *TaPBS1*/*PBS1* or *BIK1* was transiently expressed together with *pWRKY29-LUC* (Supplementary Fig. S4). Our data suggest that, in contrast to BIK1, PBS1/TaPBS1 may not play essential roles in plant PTI signaling, although both of them retain some PTI-related characteristics, like the flg22-induced phosphorylation, and the transcript levels of TaPBS1 were significantly induced when 10-day-old wheat seedlings were treated with flg22 for 60 min (Supplementary Fig. S5).

## Discussion

Activation of plant ETI relies on intracellular R proteins that detect pathogenic effectors either directly or indirectly. As for the indirect recognition manner, R proteins detect modifications of host proteins: either a modification of the effector’s host target protein that is called a “guardee”, or a modification of the host protein called a “decoy” that mimics the effector’s host target^34^. In *Arabidopsis*, PBS1 and a number of PBL proteins, such as BIK1, PBL1, PBL2, associate with, and act directly downstream of immune receptors, such as FLS2. Moreover, many of them could be cleaved by AvrPphB, allowing the pathogen to suppress host immune signaling^19^. However, only the cleavage of PBS1 by AvrPphB could be recognized by the NBS-LRR protein RPS5. It was proposed that the SEMPH motif in a C-terminal loop of PBS1 is a key specificity determinant that enables RPS5 to distinguish PBS1 from closely related kinases^24^. Therefore, PBS1 may serve as a “decoy” by mimicking true virulence targets, such as BIK1, and the SEMPH motif confers the specificity of this “decoy” function^19, 24^. However, the mechanism underlying the requirement of the SEMPH motif in RPS5 activation is not well understood. A Comparative study of a gene and its homolog from other species may help better understand its functioning mechanism. Therefore, we characterized a PBS1 homolog in wheat, TaPBS1, and performed a comparative study between TaPBS1 and PBS1. TaPBS1 shows auto-phosphorylation activity *in vitro* and is localized to the PM, in a manner dependent on an N-terminal *S*-acylation signal (Figs 4 and 5). It has the GDK motif and thus is cleaved by AvrPphB (Figs 1a and 2a). However, despite the retention of AvrPphB-mediated cleavage and the RPS5 CC association (Figs 2 and 3), co-expression of TaPBS1, AvrPphB and RPS5 could not give rise to HR in *N*. *benthamiana* (Supplementary Fig. S2). TaPBS1 harbors a STRPH motif instead of a SEMPH motif. A sequence alignment clearly shows that not a SEMPH motif but a STRPH motif is shared by PBS1 homologs of various plant species (Fig. 1a). Of wheat PBS1 homologs, only TaPBS1, TuPBS1 or AetPBS1 has the STRPH motif, and TaPBS1 is most closely related to *Arabidopsis* PBS1 (Supplementary Figs S1 and S6). Interestingly, RPS5-mediated HR was reconstituted when a TaPBS1 mutant carrying a SEMPH motif was co-expressed with AvrPphB and RPS5 (Fig. 6a). These findings demonstrate the importance of the SEMPH motif in determining the species-specific “decoy” function of PBS1. As the only difference between the SEMPH motif and the STRPH motif is the “EM” versus “TR”, our work points to the importance of “EM” in the SEMPH motif. Furthermore, using a *pbs1* protoplasts-based HR assay system developed in this study, we found that a negatively charged amino acid at the position of “E” in the SEMPH motif is required for recognition of PBS1 by RPS5 (Fig. 7a,b). And this would be a step forward in our understanding of how PBS1 fulfills its species-specific “decoy” function. However, the importance of “EM” or “E” for “decoy” function of PBS1 is specific to *Arabidopsis* RPS5. For PBS1 homologs from other plant species, perhaps motifs other than SEMPH are responsible for their recognition by the corresponding R proteins.

PBS1 associates with the CC domain of RPS5 at the PM prior to AvrPphB exposure^25^. The autoactivation of RPS5 is inhibited by the LRR domain that keeps RPS5 in the “off” state^25, 35^. The inhibition is thought to be mediated by physical associations between the LRR domain and the NBS domain of RPS5. When PBS1 is cleaved by AvrPphB, it is proposed that cleavage of PBS1 causes a conformation change that enables PBS1 to bind to the LRR domain of RPS5. This association causes a conformational change in the LRR domain that causes the LRR: NBS interface to open, thus activating RPS5^24, 35^. We found that a negatively charged amino acid at the position of “E” in the SEMPH motif is required for recognition of PBS1 by RPS5. Therefore, upon PBS1 cleavage by AvrPphB, the LRR domain of RPS5 could sense the movement of a negatively charged amino acid that is located on a predicated exposed loop of PBS1. Consequently, the LRR-mediated inhibition is removed and RPS5 is activated.

Unlike PBS1, TaPBS1 failed to activities *Arabidopsis* RPS5 upon cleavage by AvrPphB, they still share some PTI-related characteristics. TaPBS1 undergoes the flg22-induced phosphorylation in both wheat and *Arabidopsis* protoplasts. Furthermore, the flg22-induced phosphorylation of both PBS1 and TaPBS1 in *Arabidopsis* protoplasts is dependent on BAK1 and requires their PM localization but not their kinase activity (Fig. 8a–e). In addition, the expression of TaPBS1 is induced by flg22 treatment (Supplementary Fig. S5). However, unlike BIK1, both PBS1 and TaPBS1 could not activate *pFRK1-LUC* and *pWRKY29-LUC*, two luciferase reporters each driven by the promoter of a PTI responsive gene, in *Arabidopsis* protoplasts (Supplementary Fig. S4). These results suggest that, in contrast to BIK1, PBS1/TaPBS1 may not play an essential in plant PTI signaling. This observation is consistent with the findings by Zhang *et al*.^19^. They found that, unlike *bik1*, *pbs1* mutant plants showed only marginal defects in PTI defenses. Thus, PBS1 may be a relatively ancestral protein, and may have been present prior to the separation of dicots and monocots, once being an important PTI signaling component. However, later, its roles in PTI became vestigial during evolution, serving as a “decoy” by mimicking true virulence targets, such as BIK1, to trigger ETI^19, 24^.

Recently, the approach of decoy engineering was developed and it might provide a new tool for resistance breeding. Substitution of the AvrPphB-cleavage sequence in PBS1 by the cleavage sequence of other pathogen-secreted proteases resulted into the expansion of RPS5 recognition specificity^31, 32^. Application of this strategy to wheat resistance breeding requires identification of wheat pathogen-derived proteases and their preferential cleavage motifs, but first of all, whether TaPBS1 has a “decoy” function in wheat and whether wheat has R proteins recognizing TaPBS1 need to be determined in the near future.

## Methods

### Plant materials and growth conditions

*Arabidopsis* wild-type (Col-0) and *bak1* mutant plants were grown on soil in a growth chamber at 22 °C, 60% relative humidity, and 70 μE m^−2^ s^−1^ light with a 12 h photoperiod for 4 weeks for protoplasts isolation. *Nicotiana benthamiana* plants were grown under the same conditions for 4 weeks before use in gene transient expression experiments (HR assays and protein subcellular localization assays). Wheat seedlings were grown on soil in a growth chamber at 25 °C and 60% relative humidity in the dark for 10 days before protoplasts isolation.

### RNA extraction, cDNA synthesis and real-time quantitative PCR

Total RNA was extracted from 10-d-old wheat leaves using TRIzol (TransGen Biotech) following the manufacturer’s instructions. cDNA was synthesized in a 20 μL reaction using 1 mg of DNase I-treated total RNA by a reverse transcription system (TransGen Biotech), and it was then used for *TaPBS1* cloning or for real-time PCR amplification. Real-time PCR reactions were performed on an ABI 7500. Primers are listed in Supplementary Table S1.

### Isolation of *TaPBS1* gene from wheat cultivar KN2009

To identify a putative PBS1 homolog from wheat, we performed a BLASTP search in the NCBI non-redundant protein database using PBS1 as a query sequence. The top hit was a putative protein serine/threonine kinase (GenBank accession no. ADP09025.1). To isolate the CDS of this gene from wheat, we performed reverse transcription-PCR (RT-PCR) with a pair of primers designed based on the ADP09025.1 sequence. The sequence of the full-length *TaPBS1* cDNA was obtained by 5′ RACE and 3′ RACE PCR. PolyATtract® mRNA Isolation Systems III with Magnetic Stand (product Z5300, Promega Corporation) was used for mRNA enrichment, then the 5′ and 3′ RACE PCR were conducted using 5′-Full RACE Kit (D315, TaKaRa) and 3′-Full RACE Core Set Ver 2.0 (D314, TaKaRa). To clone the full-length *TaPBS1* genomic DNA, the primers were designed based on the 5′ and 3′ UTR of *TaPBS1* cDNA. Total DNA extracted from 10-d-old wheat seedlings using the CTAB method was used as template. *TaPBS1* gene information was submitted to NCBI (accession NO. KY583249). The CDSs of additional *PBS1* homologs in wheat genome were isolated from the ancestor species *Triticum urartu* or *Aegilops tauschii*.

### Plasmid construction

*Arabidopsis BIK1* and *PBS1* constructs were reported previously^18, 33^. *Arabidopsis RPS5* was amplified from Col-0 cDNA, *AvrPphB* was amplified from *P*. *syringae* pv. DC3000 (*AvrPphB*) genomic DNA^36^, and all of them were cloned into a plant expression vector with an HA, FLAG, or GFP epitope tag at the C terminus. The RPS5 coiled-coil (CC) domain was amplified as described by Qi *et al*.^35^. *TaPBS1* was also subcloned into a modified pMAL-c2 fusion protein expression vector (New England Biolabs). For HR assays in *N*. *benthamiana*, *TaPBS1*, *TaPBS1* ^*SEMPH*^, *PBS1*, *AvrPphB*, and *RPS5* were subcloned into a pER8 vector and fused to a C-terminal HA, FLAG, or Myc tag under the control of an XVE-inducible promoter^37^. All the primers used are shown in Table S1. The transient expression in *N*. *benthamiana* was confirmed by Western blot analysis with the corresponding antibodies.

### Recombinant protein expression and *in vitro* phosphorylation assays

Recombinant MBP-PBS1, MBP-TaPBS1, and MBP-BIK1 fusion proteins and mutant variants were purified from *Escherichia coli* by affinity chromatography using amylose resin (New England Biolabs) following the manufacturer’s instructions. *In vitro* kinase reactions were performed as described^38^. The protein phosphorylation was detected by an anti-phospho-Thr antibody (Cell Signaling).

### Gene transient expression in *Arabidopsis* and wheat protoplasts

*Arabidopsis* protoplasts were isolated and transfected with plant expression vectors as previously described^39^. Wheat protoplasts were isolated using a method similar to that of *Arabidopsis* using 10-d-old dark-grown wheat leaves. After transfection, protoplasts were incubated for 8 h and treated with the indicated elicitor and then used for protein mobility shift assays, co-IP assays, or subcellular localization assays. For mobility shift assays, proteins were separated on SDS-PAGE and then analyzed using Western blotting with indicated antibodies. For LUC reporter activity assay, UBQ10-GUS was co-transfected as an internal transfection control, and the promoter activity was presented as LUC/GUS ratio.

### CIP treatment

Calf intestinal alkaline phosphatase (CIP) was purchased from New England BioLabs, and the transiently expressed TaPBS1-FLAG proteins were incubated with CIP at 37 °C for 1 h. Proteins were separated with SDS/PAGE, and TaPBS1 was detected by Western blot analysis with an anti-FLAG antibody.

### Co-immunoprecipitation (co-IP) assays

Co-IP assays were performed as described previously^18^.

### Plant cell death Assays

Transient expression assay and HR assay in *N*. *benthamiana* were performed as described previously^25, 40^. *Agrobacterium tumefaciens* GV3101 strains carrying the various XVE-inducible constructs were grown and prepared for *N*. *benthamiana* inoculation. *Agrobacterium* cultures were resuspended in 10 mM MgCl_2_ at an OD_600_ = 0.6. Bacterial suspensions were infiltrated into leaves of 4-week-old *N*. *benthamiana*. Plants were then sprayed with 10 μM estradiol 30 h after *Agrobacterium* infiltration. Samples for protein extractions were collected 4 h after estradiol application. For HR assays, leaves were imaged for hypersensitive phenotypes 20 h after estradiol application. For quantification of HRs induced in *N*. *benthamiana* leaves, the biomass loss rate was measured for the infiltrated *N*. *benthamiana* leaves 36 h after estradiol application. Leaf disks of 0.5 cm in diameter were excised from leaves transiently expressing the genes related with AvrPphB-mediated HR, the same number of leaf disks were excised from control leaves. The biomass loss during HR was determined by subtracting the biomass of the latter from that of the former. Then the biomass loss rate was calculated.

For the cell death assays in protoplasts, Actin-HA was expressed together with TaPBS1/PBS1 and AvrPphB. The protoplasts were incubated and then harvested for Western blot analysis. PCD in protoplasts was determined by the repression of Actin-HA expression.

### Confocal microscopy

Confocal laser scanning microscopy was performed with a LEICA TCS SP8 AOBS inverted confocal microscope (Leica Microsystems). GFP was excited at 488 nm using an argon laser. Fluorescence emission was collected between 500–540 nm. Chloroplasts auto fluorescence was observed at 540 to 700 nm. RFP was excited at 552 nm using an argon laser. For subcellular localization assays with *N*. *benthamiana* leaves, confocal laser scanning microscopy was performed 48 h after infiltration. For analysis of plasmolyzed *N*. *benthamiana* epidermal cells, leaves infiltrated with *A*. *tumefaciens* containing the *TaPBS1-GFP* construct were mounted in 1 M sucrose before microscopy, as described previously^41^. Images were processed using Leica Application Suite Advanced Fluorescence (LAS AF) Version 4.3.

### Phylogenetic analyses and multiple alignment of PBS1 protein and its homologs

Multiple alignment of protein sequences was performed using ClustalW2^42^. The bootstrapped phylogenetic trees of full-length protein sequences were constructed using MEGA5.0.3 with the neighbor-joining method^43^.

## Electronic supplementary material

Supplementary Information
