# Supplementary material for: Comparative study of Arabidopsis PBS1 and a wheat PBS1 homolog helps understand the mechanism of PBS1 functioning in innate immunity
Source: Sci Rep. 2017 Jul 14;7:5487. doi: 10.1038/s41598-017-05904-x (PMC5511198; doi:10.1038/s41598-017-05904-x)
Supplement: Supplementary file 1 — Supplementary Information [file 41598_2017_5904_MOESM1_ESM.pdf]

Comparative study of *Arabidopsis* PBS1 and a wheat PBS1 homolog helps understand the mechanism of PBS1 functioning in innate immunity

Jianhang Sun, Guozhong Huang, Fenggui Fan, Shuangfeng Wang, Yingying Zhang, Yufang Han, Yanmin Zou, Dongping Lu*

```
TaPBS1  MGCFPCFDSGSDGELLYPKQGGGGGGNGTGGRTVSAASSSGVGAREERPMVPPRVEKLPAGAEKARAKGNAGMKELSDLR 80
TuPBS1  MGCFPCFDSGSDGELLYPKQGGGGGGNGTGGRTVSAASSSGVGAREERPMVPPRVEKLPAGAEKARAKGNAGMKELSDLR 80
AetPBS1 MGCFPCFDSGADGELLYPKQGGGGGGNGTGGRTVSAASSSGVGAREERPMVPPRVEKLPAGAEKARAKGNAGMKELSDLR 80

TaPBS1  DANGNVLSAQTFTFRQLTAATRNFREECFIGEGGFGRVYKGRLDGGQVVAIKQLNRDGNQGNKEFLVEVLMLSLLHHQNL 160
TuPBS1  DANGNVLSAQTFTFRQLTAATRNFREECFIGEGGFGRVYKGRLDGGQVVAIKQLNRDGNQGNKEFLVEVLMLSLLHHQNL 160
AetPBS1 DANGNVLSAQTFTFRQLTAATRNFREECFIGEGGFGRVYKGRLDGGQVVAIKQLNRDGNQGNKEFLVEVLMLSLLHHQNL 160

TaPBS1  VNLVGYCADGEQRLLVYEYMPLGSLEDHLHDLPPDKEPLDWNTRMKIAAGAAKGLEYLHDKAQPPVIYRDFKSSNILLGD 240
TuPBS1  VNLVGYCADGEQRLLVYEYMPLGSLEDHLHDLSPDKEPLDWNTRMKIAAGAAKGLEYLHDKAQPPVIYRDFKSSNILLGD 240
AetPBS1 VNLVGYCADGEQRLLVYEYMPLGSLEDHLHDLPPDKEPLDWNTRMKIAAGAAKGLEYLHDKAQPPVIYRDFKSSNILLGD 240

TaPBS1  DFHPKLSDFGLAKLGPVGDKSHVSTRVMGTYGYCAPEYAMTGQLTVKSDVYSFGVVLLELITGRKAIDSTRPHGEQNLVS 320
TuPBS1  DFHPKLSDFGLAKLGPVGDKSHVSTRVMGTYGYCAPEYAMTGQLTVKSDVYSFGVVLLELITGRKAIDSTRPHGEQNLVS 320
AetPBS1 DFHPKLSDFGLAKLGPVGDKSHVSTRVMGTYGYCAPEYAMTGQLTVKSDVYSFGVVLLELITGRKAIDSTRPHGEQNLVS 320

TaPBS1  WARPLFNDRRKLPKMADPGLQGRYPMRGLYQALAVASMCIQSEAASRPLIADVVTALSYLASQIYDPNAIHASKKAGGDQ 400
TuPBS1  WARPLFNDRRKLPKMADPGLQGRYPMRGLYQALAVASMCIQSEAASRPLIADVVTALSYLASQIYDPNAIHASKKAGGDQ 400
AetPBS1 WARPLFNDRRKLPKMADPGLQGRYPMRGLYQALAVASMCIQSEAASRPLIADVVTALSYLASQIYDPNAIHASKKAGGDQ 400

TaPBS1  RSRVSDSGRTLLKNDEAGSSGHKSDRDDSPREPPPGILNDRERMVAEAKMWGANLREKTRAAANAQGSLDSPTET      475
TuPBS1  RSRVSDSGRTLLKNDEAGSSGHKSDRDDSPREPPPGILNDRERMVAEAKMWGANLREKTRAAANAQGSLDSPTET      475
AetPBS1 RSRVSDSGRTLLKNDEAGSSGHKSDRDDSPREPPPGILNDRERMVAEAKMWGANLREKTRAAANAQGSLDSPTET      475
```

Figure S1. Alignment of the deduced amino acid sequences of *TaPBS1*, *TuPBS1* and *AetPBS1*. The CDS of a *PBS1* homolog from the progenitor species of hexaploid wheat, *Triticum Urartu* or *Aegilops tauschii* was isolated. Alignment of the deduced amino acid sequences of these genes was performed. The STRPH motif was highlighted in a red box.

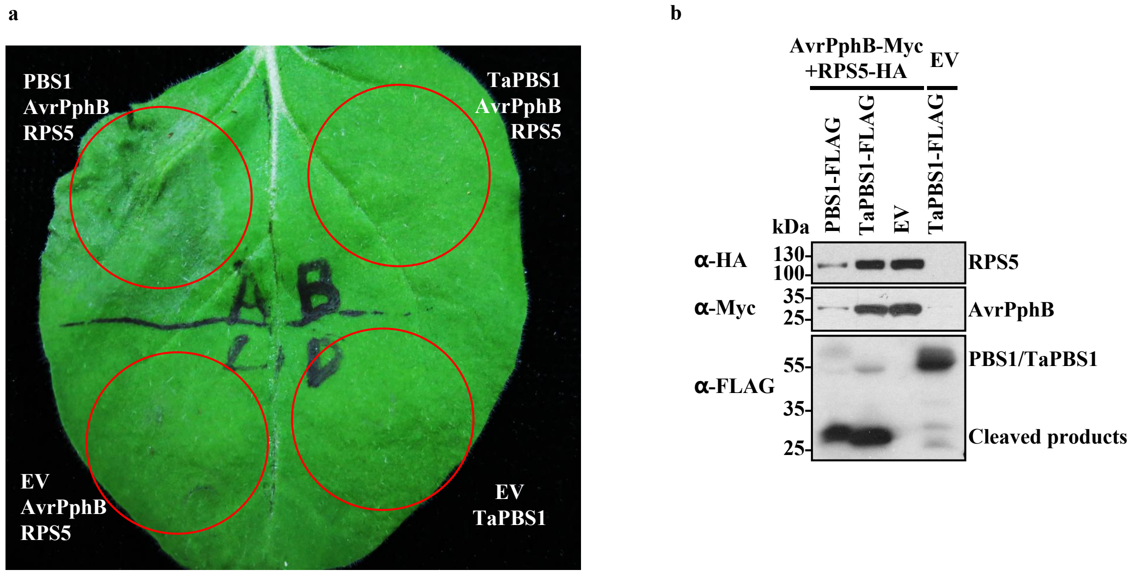

Figure S2. TaPBS1 could not activate *Arabidopsis* RPS5. (a) TaPBS1 did not activate RPS5 in *N. benthamiana*-based HR assays. PBS1 or TaPBS1 was co-expressed with RPS5 and AvrPphB in *N. benthamiana*. Plants were sprayed with 10 μM estradiol 30 h after *Agrobacterium* infiltration. Pictures were taken 20 h after protein induction. "EV" stands for empty vector. (b) The protein expression control for the *N. benthamiana*-based HR assays. The XVE-induced protein expression in *N. benthamiana* was analyzed by Western blotting with the corresponding antibodies.

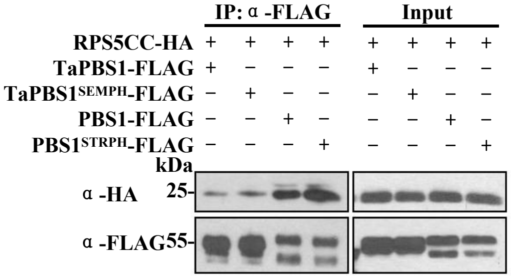

Figure S3. Change of the STRPH/SEMPH motif to the SEMPH/STRPH motif in TaPBS1/PBS1 could not affect its association with the RPS5 CC domain. TaPBS1/TaPBS1^{SEMPH}/PBS1/PBS1^{STRPH}-FLAG and RPS5-CC-HA were co-expressed in *Arabidopsis* protoplasts, and TaPBS1/TaPBS1^{SEMPH}/PBS1/PBS1^{STRPH} was precipitated with an anti-FLAG antibody. The associated proteins were analyzed by Western blotting with an anti-HA antibody.

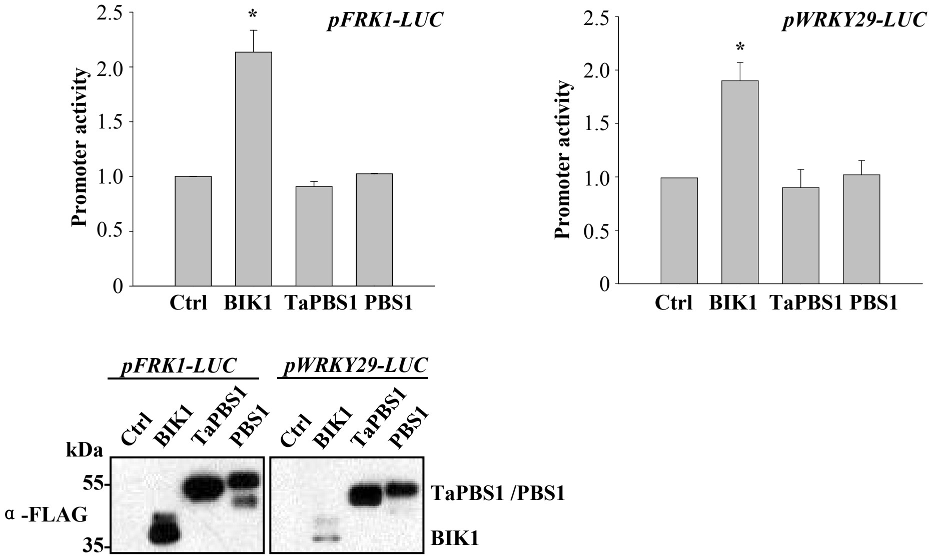

Figure S4. Expression of PBS1/TaPBS1could not activate the promoter activities of *pFRK1-LUC* and *pWRKY29-LUC* in protoplasts. Protoplasts were co-transfected with *BIK1/PBS1/TaPBS1* and *pFRK1-LUC*/*pWRKY29-LUC* and incubated for 6h. *UBQ10-GUS* was included as a transfection control, and the promoter activity was determined by a LUC:GUS ratio. Asterisk indicates significant differences with $P < 0.05$ (*t*-test). The lower panel shows the transient expression of BIK1/PBS1/TaPBS1-FLAG in protoplasts.

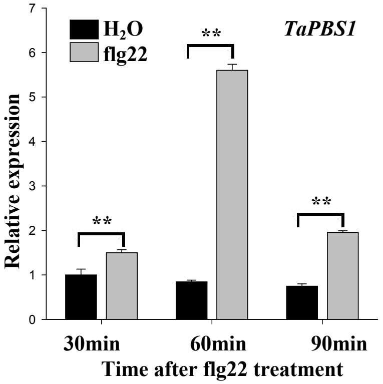

Figure S5. The TaPBS1 transcript level is up-regulated upon flg22 treatment. The 10-d-old wheat leaves were cut to 1 cm length pieces and incubated overnight in water, and then were treated with 5 μM flg22 or water for the indicated times. RNA was extracted and real-time qRT-PCR analysis was performed. The reuslts are shown as means ± SEs from three replicates. Asterisks indicate significant differences with $P < 0.01$ (*t*-test).

```
TaPBS1      TGRKA--IDSTRPHGEQNLVS
CDM82645.1  TGRRA--LDSNRPREEQDLVS
ABY59656.1  TGRRA--IDTTKPTREQILVH
ABY59655.1  TGRRA--IDTTKPTREQILVH
ABG68032.1  TGRRA--IDTTKPTREQILVH
ABG68041.1  TGRRA--IDTTKPTREQILVH
CDM84543.1  TGRRA--IDPTKPTEEQVLIH
CDM83073.1  SGRRS--MDKNRPNGEHNLVE
AGU99598.1  SGRRA--IDKNRPQGEHNLVE
CDM83130.1  TGRKA--VDISRPKGQQFLTE
CDM82475.1  TGRDP--VDYGRPANEVNLVD
CDM83128.1  TGRDP--VNYSRPANEVHMVE
CDM84372.1  CGQRP--VKQNANGDRVMLVD
AGY79316.1  TGRRNSGSYNTEQDVD--LLN
CDM84608.1  CGRRP--VSNGPQDSHEMLVD
ADK62369.1  CGRRP--IEEDEGNNRVMLVD
ACL36475.1  CGRRP--IEEDGGNNRVMLVD
ADK62373.1  CGRRP--IEEDEENNRVMLVD
ACN41357.1  CGRRP--IERGERNTPVVMID
CDM84469.1  CGRQP--SEPTAEPSKARLVP
CDM86040.1  CGRRPLLVLRGNEACTMHLVQ
CDM85457.1  SGRPA--LDLAEPSGMVLVTD
```

Figure S6. Multiple sequence alignment of the STRPH-containing regions of the potential wheat PBL homologs. The STRPH motif regions are highlighted in a box.

Table S1. Primers used for cloning and Real-time RT-PCR.

Gene, Mutant, and Vector	Primer Sequence (5'-3', for the underlined segments)		Restriction site	Use
	Forward (or LP)	Reverse (or RP)		
q-TaPBS1	CCTGAAGAATGATGAGGCAG	CCGGTTTCAGTTGGAGAATC		real-time PCR
q-TaGAPDH	TTAGACTTGCGAAGCCAGCA	AAATGCCCTTGAGGTTTCCC		real-time PCR
pHBT-TaPBS1-FLAG	CCGGATCCATGGGTTGCTTCCCGTGCT	GAAGGCCTTCCGGTTTCAGTTGGAGAATCG	*BamHI/StuI*	cloning
pHBT-TaPBS1 $^{G2AC3/6A}$*-FLAG*	CGGGATCCATGGcTgcCTTCCCGgcCTTCGATTCGGGC	GAAGGCCTTCCGGTTTCAGTTGGAGAATCG	*BamHI/StuI*	point mutation
pHBT-TaPBS1 ^{K132A}*-FLAG*	GTTGTTGCTATAgcGCAGCTCAATAG	CTATTGAGCTGCgcTATAGCAACAAC		point mutation
pHBT-BIK1 ^{K105N}*-FLAG*	GTCATCGCCGTTgcAGCGCTTAACCAAGAA	TTCTTGGTTAAGCGCTgcAACGGCGATGAC		point mutation
pHBT-PBS1 ^{K115N}*-FLAG*	GGTTGTTGCTGTTAAcCAACTAGACAGGAATG	CATTCCTGTCTAGTTGGgTTAACAGCAACAACC		point mutation
pHBT-AvrPphB-Myc	CGGGATCCATGAAAATAGGTACGCAGGCC	AAGGCCTCGAAACTCTAAACTCGTTTACG	*BamHI/StuI*	cloning
pHBT-RPS5-HA	CGGGATCCATGGGAGGTTGTTTCTCTG	AAGGCCTTGTTTCTCTCCACCGCCACCTGG	*BamHI/StuI*	cloning
pHBT-RPS5-CC-HA	CGGGATCCATGGGAGGTTGTTTCTCTG	AAGGCCTACCGTACAGACCCAAAATCC	*BamHI/StuI*	cloning
per8-AvrPphB-Myc	CCGCTCGAGATGAAAATAGGTACGCAGGCCACC	GACTAGTGAGGTCCTCCTCGGAGATGAGCTTCTGCTCAGG	*XhoI/SpeI*	cloning
per8-RPS5-HA	AGGCGCGCCATGGGAGGTTGTTTCTCTG	AATCAAGGGCCCTCAAGCGTAGTCTGGAACG	*AscI/ApaI*	cloning
per8-PBS1-FLAG	CCGCTCGAGATGGGTTGTTTCTCGTG	GACTAGTCTGCAGTCACTTGTCATCGTCG	*XhoI/SpeI*	cloning
per8-TaPBS1-FLAG	CCGCTCGAGATGGGTTGCTTCCCGTGC	GACTAGTCTGCAGTCACTTGTCATCGTCG	*XhoI/SpeI*	cloning
per8-TaPBS1 ^{T310ER311M}*-FLAG*	ATTGACAGCgagatgCCTCATGGGGAACAAAACCTC	AGGcatctcGCTGTCAATGGCCTTCCGG		point mutation
T-TaPBS1-5' outer	CATGGCTACATGCTGACAGCCTA	TGGTGCTGTCAATGGCCTTCCG		5' RACE
T-TaPBS1-5' inner	CGCGGATCCACAGCCTACTGATGATCAGTCGATG	CACCAGAAGGCGTTGCTCTCC		
T-TaPBS1-3 outer	AGCAGCTCAATAGGGATGG	TACCGTCGTTCCACTAGTGATTT		3' RACE
T-TaPBS1-3' inner	AGCTGGTGCTGCTAAAGGGCTG	CGCGGATCCTCCACTAGTGATTTCACTATAGG		
T-TaPBS1 Genomic DNA	GCTCTGTCCGGCACGAATCC	TTCCGAGAATTTACAGGTACG		cloning
pHBT-GFP-FLAG	CGGGATCCATGGTGAGCAAGGGCGAGGAG	AAGGCCTCTTGTACAGCTCGTCCATGCCGA	*BamHI/StuI*	cloning
pHBT-BSK1-RFP	AAGGCCTATGGCCTCCTCCGAGGACG	AACTGCAGTTAGGCGCCGGTGGAGTGG	*StuI/PstI*	cloning
